# Pre-transplant Thymic Function Predicts Is Associated With Patient Death After Kidney Transplantation

**DOI:** 10.3389/fimmu.2020.01653

**Published:** 2020-07-31

**Authors:** Cécile Courivaud, Jamal Bamoulid, Thomas Crepin, Emilie Gaiffe, Caroline Laheurte, Philippe Saas, Didier Ducloux

**Affiliations:** ^1^Inserm, UMR1098, Federation Hospitalo-Universitaire INCREASE, Besançon, France; ^2^Univ. Bourgogne Franche-Comté, Faculté de Médecine et de Pharmacie, LabEx LipSTIC, Besançon, France; ^3^Structure Fédérative de Recherche, SFR FED4234, Besançon, France; ^4^CHU Besançon, Department of Nephrology, Dialysis, and Renal Transplantation, Besançon, France; ^5^CHU Besançon, CIC Biothérapie, INSERM CIC1431, Besançon, France; ^6^EFS Bourgogne Franche-Comté, Plateforme de Biomonitoring, CIC 1431/UMR1098, Besançon, France

**Keywords:** kidney transplantation, thymus, death, immune senescence, cancer

## Abstract

Accelerated thymic involution is a main feature of end-stage renal disease (ESRD)-associated immune senescence. Recent evidences suggest that ESRD-associated immune senescence is associated with adverse outcomes in dialysis patients. However, no study focused on the association between pre-transplant thymic function and patient survival after transplantation. We conducted a prospective, multicenter study to assess whether pre-transplant thymic function measured by recent thymic emigrants (RTE) may predict death after first kidney transplantation. Results were tested in a validation cohort. Nine hundred and sixty-seven incident kidney transplant recipients were included in the prospective study. Mean follow up was 5.1 + 2.9 years. Eighty two patients (8.5%) died during follow up. Lower RTE levels were associated with a higher risk of death (2.53; 95%CI, 1.54–4.39 for each decrease of 1 log in RTE; *p* < 0.001). Cancer-related death was particularly increased in patients with low RTE levels (4.23; 95%CI, 1.43–12.13; *p* = 0.007). One hundred and thirty-six patients having received a first kidney transplantation were included in the validation cohort. Lower TREC levels were associated with higher risk of death (1.90; 95%CI, 1.11–3.51 for each decrease of 1 log in RTE; *p* = 0.025). RTE were not associated with death-censored graft loss. Pre-transplant thymic function is strongly associated with death after transplantation. Attempt to reverse ESRD-related thymic loss may prevent premature death.

## Introduction

Involution of the thymus with age is associated with a decline in naïve T cell output. This contributes to the reduction in T cell diversity observed in elderly subjects and to a decreased ability of the immune system to generate antigen specific responses against pathogens and vaccines. Altered thymic activity is considered the founding event of the decline of immune function in older individuals (1). Many studies reported accelerated thymic loss in patients with chronic kidney diseases (2, 3), and thymic involution is now well-described as a part of ESRD-associated immune senescence which also includes T cell exhaustion, shortening of telomeres, and inflammation (2, 3). Recent evidences suggest that end-stage-associated immune senescence is associated with adverse outcomes in ESRD patients (3).

Concordant reports also indicate that ESRD-associated immune senescence affects graft outcomes after kidney transplantation. We recently reported that a preserved thymic function was associated with higher risk of acute rejection in ATG-treated kidney transplant recipients (4). By contrast, expansion of terminally differentiated CD8+ T cell seems to be associated with a low incidence of acute rejection (5, 6). However, loss of CD28 on peripheral T cells decreases the risk for early acute rejection after kidney transplantation (7). However, very few data concern the effects of ESRD-associated immune senescence on patient outcomes after kidney transplantation. We reported that pre-transplant immune risk profile was a risk factor for post-transplant infections (6), but this result was not confirmed in another study (8). Thymic function is essential for responses against infectious pathogens and immune surveillance against cancer. We also observed in a small retrospective cohort that pre-transplant thymic function predicts cancer after transplantation (9). Our group also reported studies showing associations between thymic function and infections (6) and CD4 T cell lymphopenia and cardiovascular events and death (9). Thymic output can be assessed by either recent thymic emigrants (RTE) or T cell receptor excision circle (TRECs) measurement (10). Results are reliable and well-correlated (4, 10).

Nevertheless, to date, no study specifically focused on the association between pre-transplant thymic function and strong outcomes such as patient survival after transplantation. To test this hypothesis, we conducted a large prospective, multicenter study to assess whether pre-transplant thymic function may predict death after kidney transplantation. The results were also tested in an independent retrospective validation cohort.

## Subjects and Methods

### Study Design and Populations

#### Prospective Cohort

Research has been conducted in 1,062 consecutive RTR included in the ORLY-EST study with at least 1-year follow-up (transplant before April 2018). In order to study a more homogeneous population, we only considered patients having received a first kidney transplantation (*n* = 967). Briefly, ORLY-EST is an observational prospective study including incident RTR in seven French transplant centers (Besançon, Clermont-Ferrand, Dijon, Kremlin-Bicêtre, Nancy, Reims, Strasbourg). The main objective of this study is to describe interactions between immune status and post-transplant atherosclerosis. For each patient, blood samples were collected at time of transplantation and 1 year after. Sample collection was performed after regulatory approval by the French ministry of health (agreement number # DC-2008-713, June 11th 2009). The ethic committee of Franche-Comté study has approved the study (2008). Patients enrolled in the ORLY-EST study gave their written informed consent. Clinical data were prospectively collected.

Among these 967 RTR, 290 patients (30%) had received T cell depleting ATG therapy and 677 (70%) had received non-depleting α-CD25 mAb therapy. Calcineurin inhibitors (CNI) and Mycophenolate Mofetil (MMF) were widely used as immunosuppressive regimen. All the transplants were performed with a negative CM cross-match. For details, see the Methods in the supporting information section.

Cytomegalovirus (CMV) prophylaxis was given according to each center practice. Almost all CMV-exposed patients received valganciclovir for 3 months. All CMV-naïve patients having received a CMV positive kidney received valganciclovir for 3 or 6 months. All patients received Pneumocystis antimicrobial prophylaxis with trimethoprim-sulfamethoxazole for at least 6 months.

Database was closed for analysis on 30th April 2019. Minimum follow-up was 1-year post-transplant.

#### Retrospective Validation Cohort

We used a previously published cohort of patients. Briefly, all of the patients who underwent transplantation in our center between March 1999 and November 2004 (*n* = 177) have been included in a study on pre-transplant determinants of post-transplant CD4 T cell lymphopenia (9).

Pre-transplant TREC levels were available in only 157 patients and 136 had received a first kidney transplant.

All of these patients received the same maintenance immunosuppressive treatment, including tacrolimus, azathioprine, and steroids.

These patients served as a validation cohort.

### Confounding Factors

Age, gender, body mass index, diabetes, dyslipidemia, hypertension, smoking habit, past history of cardiovascular events (CVE), previous neoplastic history, and chronic lung disease were analyzed as covariates. Dialysis mode (none, hemodialysis, or peritoneal dialysis), and its duration prior to transplantation were also recorded. HLA mismatches were recorded for HLA-A, -B, and -DR loci. Other relevant immunological parameters such as, pre-transplant panel reactive antibodies (PRA) (0 *vs*. positive PRA at any level), and transplant type (living/deceased) were analyzed as covariates. Cold ischemia time, donor age, and presence of delayed graft function were also considered. Methods of assessment and definitions of these variables have been previously described in details (10).

### Lymphocyte Subsets

#### T Cell Immunophenotypic Analysis

Absolute numbers of CD4^+^ and CD8^+^ T cells were determined on fresh samples by a single platform flow cytometry approach using TetraCXP® method, Flow-Count® fluorospheres and FC500 cytometer (Beckman Coulter, Villepinte, France) according to manufacturer's recommendations. PBMCs were isolated by density gradient centrifugation (Pancoll, Pan-Biotech GmBH Aidenbach, Germany) and cryopreserved. After thawing, PBMCs were washed twice in RPMI 1640 + GlutaMAX™-I medium (Invitrogen, Cergy-Pontoise, France) containing 10% fetal calf serum (Invitrogen), thereafter referred as complete medium. Cells were stained with the following conjugated antibodies directed against: CD3, CD4, CD8, CD31, CD45RA, CD45RO, CD16, CD19, and CD56. Cell debris and doublets were excluded on the basis of side vs. forward scatter. Cells were analyzed on a FACS CANTO II cytometer (BD Biosciences) using FACS Diva (BD Biosciences) software.

Recent thymic emigrants (RTE) were defined as CD45RA^+^CD31^+^CD4^+^ T cells (9) (Figure S1). Data were analyzed by considering the percentage of RTE among CD4^+^ T cells (RTE frequency or RTE%) and the absolute numbers of circulating RTE/mm^3^. Pre-transplant RTE were considered for analysis.

### T-Cell Receptor Excision Circle (TREC)

The signal-joint TRECs were quantified in peripheral blood mononuclear cells using real-time quantitative PCR on LightCycler (Roche Diagnostics, Meylan, France), as described previously (9). A series of standard dilutions of a plasmid containing the signal-joint breakpoint was used to quantify TRECs in each patient and control DNA sample. Cycle threshold was assessed using the second derivative method with the LightCycler 3.5.3 software (Roche Diagnostics). Each DNA sample was run in duplicate. Quantification of a reference gene (GAPDH) was carried out in the same conditions. Values were normalized for the genomic copy number using GAPDH quantification and corrected for the percentage of CD3^+^ cells in peripheral blood mononuclear cells, as described previously (11).

### Outcomes

The primary endpoint was the death in transplantation defined by patient's death with a functional graft. Causes of death were also analyzed.

### Statistical Analysis

#### Baseline Characteristics

Median (interquartile range), mean values (standard deviation), and frequency (percentage) were provided for continuous and categorical variables, respectively. Medians, means, and proportions were compared using Student's *t*-test and chi-square test (or Fisher's exact test, if appropriate), respectively. Correlations between variables were assessed by Pearson correlation coefficient.

Follow-up duration was calculated using a reverse Kaplan-Meier estimation.

Because RTE values were not normally distributed, they were either log-transformed or split in quartiles. Colinearity among biological variables were tested with a correlation matrix.

Using log rank tests on Kaplan Meier nonparametric estimates of the survival without death distribution, we selected variables with a *p* ≤ 0.20. The selected variables were included into a Cox proportional hazards model, and a backward stepwise selection process was performed, this time at a classical α = 0.05. However, the Kaplan-Meier method is known to overestimate the probabilities of event of interest when there are competing events. Here, transfer to dialysis as competing event of death provides justification for competing risk analysis (Fine and Gray model).

Results are expressed as hazard ratio (HR) and 95 % confidence interval (CI), with a *p* value testing the null hypothesis: HR = 1. Therefore, when *p* < 0.05, HR is significantly different from 1, either greater than 1 (i.e., risk of death is increased) or <1 (*i.e*., risk of death is decreased). Assumptions of Cox models (log-linearity, proportionality of risk in time) were met in this analysis.

Wald test was used to test potential interactions between variables.

## Results

### Study Population

Characteristics of the study population were depicted in Table 1. Nine hundred and sixty seven patients were included. Mean age was 52 ± 14 years and about two third of patients were male. More than ninety percent of patients received a first transplant.

**Table 1:** Demographic characteristics of the study population.

***N* = 967**	**Mean**
Age (Years)	52 ± 14
Gender (% male)	65%
Pre-transplant Dialysis	91%
Dialysis vintage (months)	40 ± 35
BMI (kg/m^2^)	25.8 ± 4.7
Pre-transplant diabetes	21%
Current smoking (%)	23%
Pre-transplant history of cancer	7%
Pre-transplant history of CVD	20%
Chronic respiratory disease	4%
Pre-transplant CMV exposure	55%

Mean follow up was 5.1 + 2.9 years.

The rate of missing data was <5% for all studied parameters.

### Pre-transplant Thymic Function

Both pre-transplant CD45RA^+^CD31^+^ CD4^+^ T cells (RTE/mm^3^) and RTE frequency (percentage of CD45RA^+^CD31^+^ CD4^+^ T cells among CD4^+^ T cells) were abnormally distributed. Median value of CD45RA^+^CD31^+^ CD4^+^ T cells (RTE/mm^3^) was 138/mm^3^ (range: 2–1,135). This T cell subset accounts for 25% (median, range: 2–67) of CD4^+^ T cells before transplantation.

Pre-transplant RTE count was inversely related to age (*r* = −0.33; *p* < 0.001) and to dialysis duration (*r* = −0.14; *p* = 0.001). Similar results were observed for RTE frequency.

RTE were split into quartiles. A number of clinical conditions differed between quartiles. Younger age (*p* < 0.001), female gender (*p* < 0.001), absence of diabetes (*p* = 0.007), and shorter dialysis duration (*p* = 0.017) were independent predictors of highest values of RTE (Table 2). A past history of cancer or cardiovascular disease was more frequently observed in patients with low RTE levels (Table 2).

**Table 2:** Clinical characteristics of the study population according to RTE quartiles.

	**Q1 (*n* = 242)**	**Q2 (*n* = 241)**	**Q3 (*n* = 242)**	**Q4 (*n* = 242)**	***p***
Age (years)	59 + 12	55 ± 13	53 ± 14	46 ± 13	<0.001
Gender (% male)	70%	68%	63%	54%	<0.001
Dialysis	94%	94%	89%	86%	<0.001
Dialysis vintage (months)	40+34	43 ± 34	42 ± 38	35 ± 30	<0.001
Diabetes	28%	24%	17%	10%	<0.001
Hypertension	82%	83%	90%	87%	0.019
Body mass index (kg/m^2^)	26.2 + 4.7	25.8 ± 4.8	25.9 ± 5.0	24.9 ± 5.1	0.541
Past history of cancer	12%	9%	6%	3%	<0.001
Past history of CVD	24%	21%	18%	12%	<0.001
Chronic respiratory disease	4%	4%	3%	4%	0.482
Percentage of Immunized patients	30%	33%	31%	30%	0.521
Pre-transplant CMV exposure	62%	61%	51%	54%	0.054
RTE (/mm^3^) Median (range)	44 [2–73]	107 [75–134]	177 [135–230]	316 [231–1,136]	<0.001

### Thymic Function and Associated Immune Profile

Pre-transplant RTE count was highly related to other CD4 T cell subsets as well as CD3 T cell count (Table S1). Associations between RTE counts and both B cell and monocytes counts were significant but weak. Matrix correlation is depicted in supplementary data (Table S1). Patients in the different quartiles of RTE differed for all studied immune cell counts except inflammatory monocytes (data not shown). Patients with low pre-transplant RTE levels had typically low CD3 T, CD4 T, CD8 T, B, and NK cell counts.

### Death

Eighty two patients (8.5%) died during follow up. Causes of death were: infection (*n* = 28, 35%), cardiovascular (*n* = 21, 25%), cancer (*n* = 19, 23%), other (*n* = 9, 10%), and undetermined (5, 7%).

Because RTE were abnormally distributed, they were studied first after log-transformation and second using quartiles of RTE.

### Log RTE

In univariate analysis, age (1.06; 95%CI, 1.04–1.08 for each increase of 1 year; *p* < 0.001), male gender (2.90; 95%CI, 1.65–5.12; *p* < 0.001), body mass index (1.08; 95%CI, 1.04–1.12 for each increase of 1 kg/m^2^; *p* < 0.001), diabetes (4.11; 95%CI, 2.64–6.32; *p* < 0.001), dialysis duration (2.09; 95%CI, 1.28–3.25 for dialysis vintage >25 months; *p* = 0.003), a past history of cardiovascular disease (1.68; 95%CI, 1.24–2.30; *p* = 0.011), and chronic respiratory failure (2.41; 95%CI, 1.45–4.01; *p* < 0.001) were associated with death.

Lower pre-transplant RTE levels were associated with a higher risk of death (4.22; 95%CI, 2.84–6.73 for each decrease of 1 log in RTE; *p* < 0.001).

CD4 and CD3 T cell counts were also associated with death (4.81; 95%CI, 2.07–13.06 for each decrease of 1 log in CD3 T cell count; *p* < 0.001, and 4.87; 95%CI, 2.17–11.56 for each decrease of 1 log in CD4 T cell count; *p* < 0.001). Nevertheless, trivariate stepwise analysis including the three T lymphocytes subsets only retains pre-transplant RTE as associated with death (5.39; 95%CI, 2.82–11.37 for each decrease of 1 log in RTE; *p* < 0.001). As a consequence, only RTE was maintained in the model.

Analyses showed significant interaction between log RTE and age (*p* < 0.001). Three tertiles of age were achieved. The influence of RTE on death risk was more important in younger patients. HR were 12.34 (95%CI, 3.05–165 for each decrease of 1 log; *p* < 0.001), 3.86 (95%CI, 1.71–8.67 for each decrease of 1 log; *p* < 0.001), and 2.01 (95%CI, 1.03–4.15 for each decrease of 1 log; *p* = 0.032), respectively in tertiles 1, 2, and 3 (Table 3). Test for interaction was significant (*p* < 0.001). No other interaction was detected and log RTE remained associated with death after adjustment for all relevant parameters (Table 4).

**Table 3:** Association between log RTE and death in different sub-populations.

	**HR**	**95%CI**	***p***
Age T1	12.34	3.05–165	<0.001
Age T2	3.86	1.71–8.67	<0.001
Age T3	2.01	1.03–4.15	0.032
ATG-	4,34	2.56–7.14	<0.001
ATG+	3.70	1.52–9.09	0.004
Dialysis duration ≤ 25 months	3.22	1.21–5.62	0.011
Dialysis duration>25 months	5.00	2.94–8.33	<0.001
Total	4.22	2.84–6.73	<0.001

*Tertiles of age*.

*T1: 18–47 years*.

*T2: 48–61 years*.

*T3: 62–84 years*.

*ATG−: patients not having received ATG*.

*ATG+: patients having received ATG*.

**Table 4:** Association between log RTE and death, unadjusted, bivariate analysis, and full adjusted.

**UNIVARIATE**	
	4.22; 95%CI, 2.84 to 6.73
**ADJUSTMENT FOR,**	
Age	3.22; 95%CI, 1.94–4.69
Gender	4.02; 95%CI, 2.51–6.33
Dialysis vintage	3.82; 95%CI, 2.58–6.28
Diabetes	3.52; 95%CI, 2.25–5.64
Previous CVD	4.18; 95%CI, 2.83–6.75
Past cancer	4.19; 95%CI, 2.75–6.71
**FULL ADJUSTMENT,**	
	2.53; 95%CI, 1.54–4.39

*For each decrease of 1 log in RTE*.

Because ATG profoundly affects RTE, the association between pre-transplant RTE and death was studied separately in patients having or not received ATG. No differential effect of RTE was observed in the two groups (Table 3 and Figures S2A,B).

In multivariate analysis, age (1.04; 95%CI, 1.02–1.06 for each increase of 1 year; *p* < 0.001), male gender (2.22; 95% CI, 1.22–4.12; *p* = 0.008), diabetes (2.50; 95% CI, 1.55–3.99; *p* = 0.001), and dialysis duration (1.59; 95% CI, 1.02–2.77 for dialysis vintage > 25 months; *p* = 0.041) were associated with early post-transplant death. Lower pre-transplant RTE levels remained associated with a higher risk of death (2.53; 95%CI, 1.54–4.39 for each decrease of 1 log in RTE; *p* < 0.001) (Table 3 and Table S2).

Similar results were obtained using RTE frequency instead of RTE absolute value (1.03; 95% CI, 1.01–1.05 for each decrease of 1% in RTE frequency; *p* = 0.003).

### Quartiles of RTE

**Figure 1:**
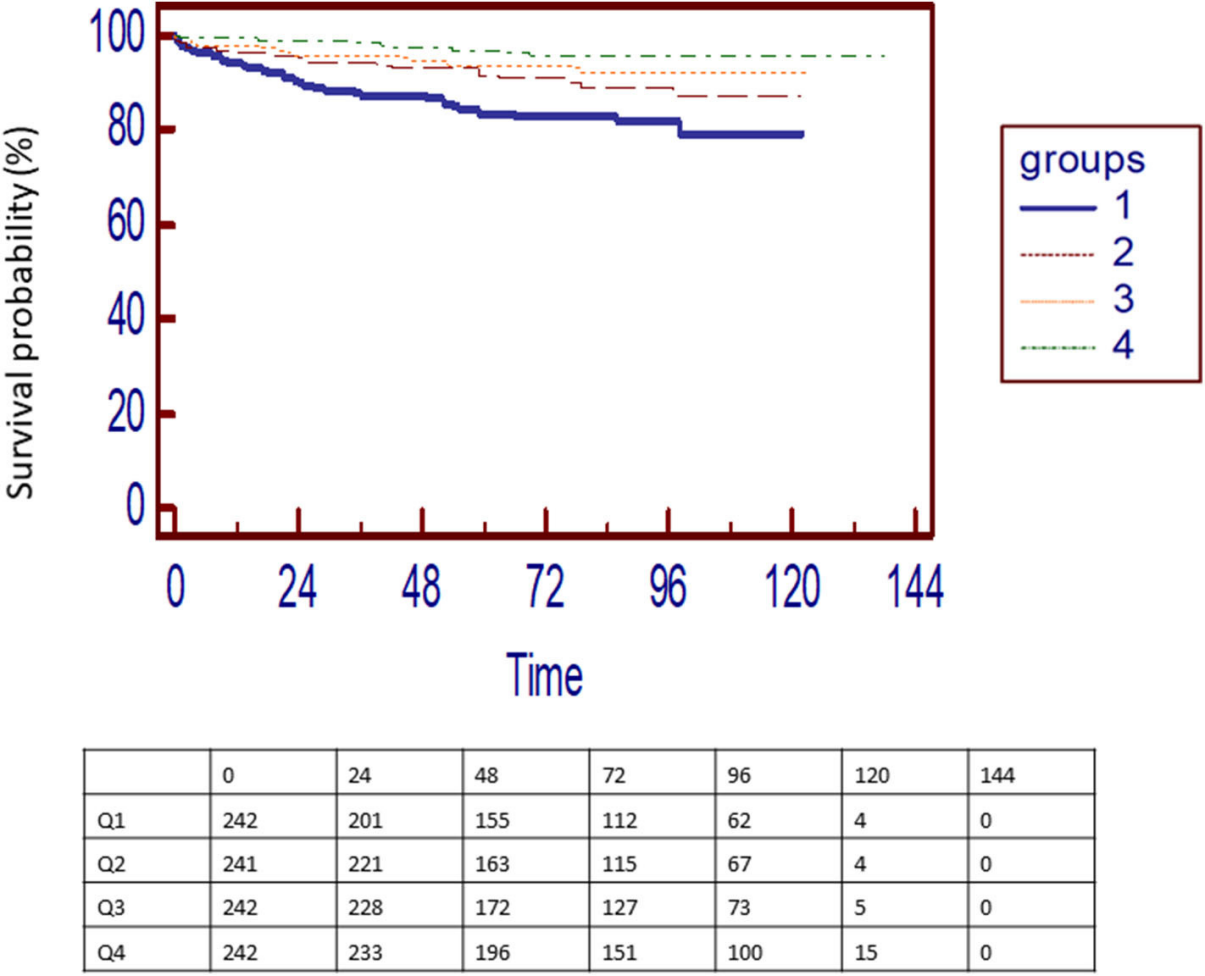
Kaplan-Meier death in transplantation-free survival curves for patients according to quartiles o RTE. Ranges of RTE: Q1 [2–74], Q2 [75–135], Q3 [136–230], and Q4 [231–1,135].

Four quartiles of pre-transplant RTE were defined (Table 2). Death rates decreased from quartile 1 to quartile 4 (Figure 1). Compared to upper quartiles (Q1) of RTE, risk of death was 0.54 (0.30 to 0.96; *p* = 0.036), 0.36 (0.21–0.66; *p* < 0.001), and 0.17 (0.11–0.35; *p* < 0.001) in Q2, Q3, and Q4, respectively.

In multivariate analysis, risk of death was 0.62 (0.36–1.05; *p* = 0.086), 0.54 (0.29–0.99; *p* = 0.048), and 0.41 (0.20–0.89; *p* = 0.021) in Q2, Q3, and Q4, respectively, compared to Q1 (*p* for trends < 0.001).

Neither RTE frequency nor RTE absolute value was associated with death-censored graft loss (Figure S3).

### Causes of Death According to Thymic Function

We analyzed causes of death in patients with pre-transplant RTE value under or above 138/mm^3^ (median value).

**Figure 2:**
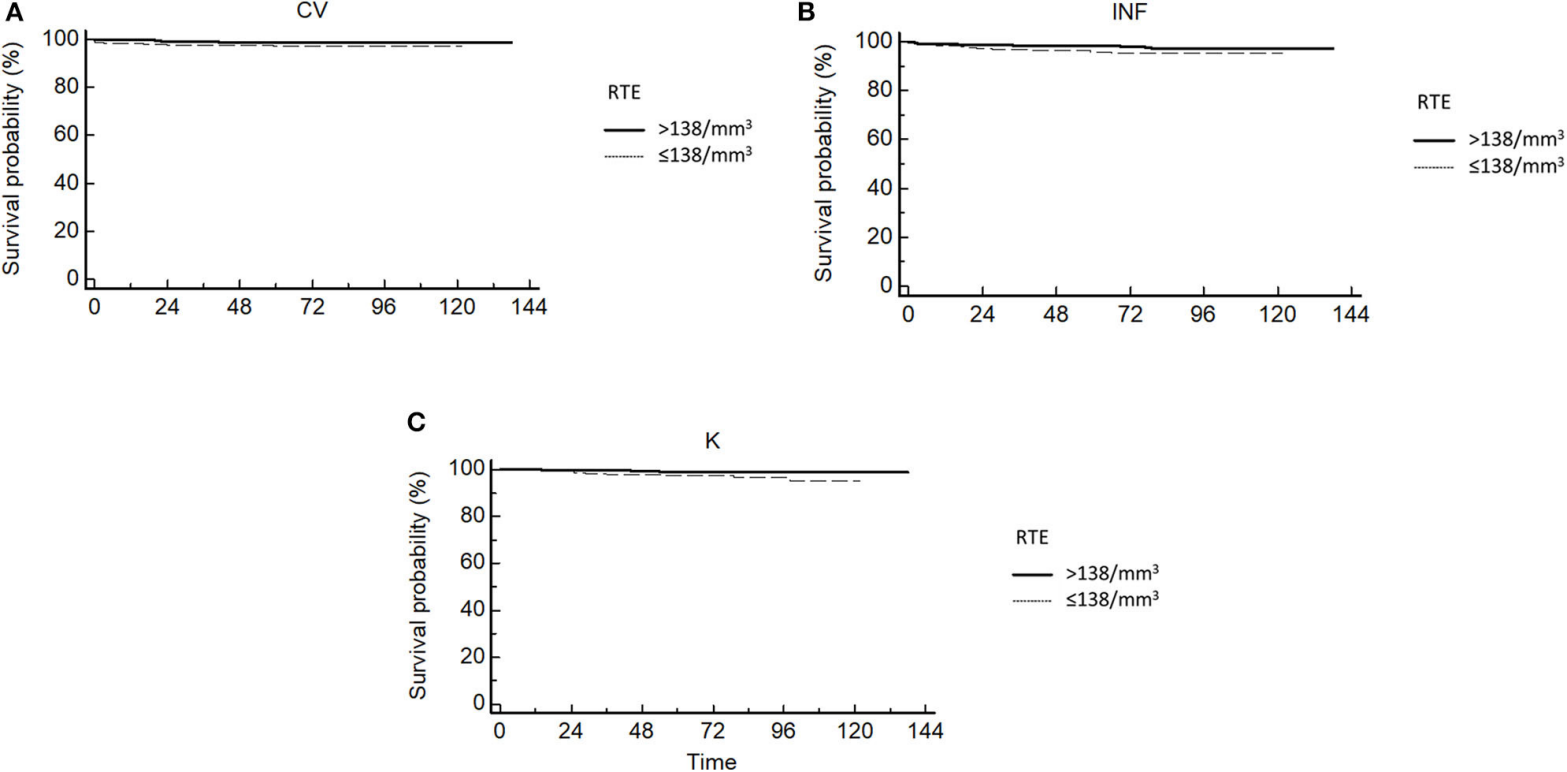
**(A)** Kaplan-Meier cardiovascular death in transplantation-free survival curves for patients according to quartiles of RTE. **(B)** Kaplan-Meier infection-related death in transplantation-free survival curves for patients according to quartiles of RTE. **(C)** Kaplan-Meier cancer death in transplantation-free survival curves for patients according to quartiles of RTE.

All causes of death were increased in patients with low RTE levels (Figures 2A–C).

RTE levels were associated with all causes of death, but after adjustment for age, only association with cancer-related death remained significant (4.66; 95%CI, 1.52–13.95 for each decrease of 1 log in RTE; *p* = 0.006). Cancer-related death was also associated with diabetes and pre-transplant history of cancer. After multivariate analysis, RTE remained strongly associated with cancer-related death (4.23; 95%CI, 1.43–12.13 for each decrease of 1 log in RTE; *p* = 0.007).

We separately studied the impact of pre-transplant RTE on death and cancer-related death in patients with and without a pre-transplant history of cancer. HR for death were very similar (0.23; 95%CI, 0.06–1.04, for each increase of 1 log in RTE; *p* = 0.057, and 0.24; 95%CI, 0.16–0.40, for each increase of 1 log in RTE; *p* < 0.001, respectively) in the two populations. Concerning cancer-related death, HR were 0.18; 95%CI, 0.05–0.72, for each increase of 1 log in RTE; *p* = 0.011, and 0.23; 95%CI, 0.06–1.21, for each increase of 1 log in RTE; *p* = 0.099), respectively in patients with a pre-transplant history of cancer and in those without.

### Cohort of Replication

One hundred and thirty-six patients were included in the validation cohort. Characteristics of the studied population have been previously reported (9) and are summarized in Table 5 and Table S3.

**Table 5:** Clinical characteristics of the replication cohort.

	**Anti-CD25 mab (39)**	**ATG (97)**	***p***
Age (years)	54 ± 14	52+14	0.082
Gender (% male)	66%	64%	0.762
Diabetes	20%	22%	0.884
Dyslipidemia	33%	35%	0.849
Hypertension	85%	84%	0.874
Body mass index (kg/m^2^)	23.8 ± 4.1	24.2 + 4.2	0.501
Percentage of Immunized patients	18%	33%	0.079
Positive CMV serology	66%	79%	0.118

Pre-transplant CMV exposure was more frequent in the validation cohort (*p* < 0.001). ATG was more frequently used in the validation cohort (*p* < 0.001) (Table S2).

27 patients (20%) died during follow up (8.7 ± 3.4 years).

As T-cell Receptor Excision Circle (TREC) values were not normally distributed, pre-transplant TREC levels were log-transformed. We observed a strong inverse correlation between TREC levels and age (r = −0.45, *p* < 0.001).

Lower log TREC were associated with higher risk of death (2.47; 95%CI, 1.32–4.22 for each decrease of 1 log in TREC; *p* < 0.001). The association remained significant after adjustment for age (1.90; 95%CI, 1.11–3.51 for each decrease of 1 log in TREC; *p* = 0.025).

### RTE 1 Year Post-transplant

RTE at 1 year were closely related to RTE at transplant (*r* = 0.508, *p* < 0.0001) (Figure S4). RTE at 1 year was weakly associated with 1-year post-transplant GFR (*r* = 0.12, *p* = 0.078). Lower one-year post-transplant RTE levels were associated with a higher risk of death (1.75; 95%CI, 1.14–2.71 for each decrease of 1 log in RTE; *p* = 0.013) (Figure S5). Adjustment for eGFR does not modify the association between 1-year post-transplant logRTE and death (1.69; 95%CI, 1.11–2.78 for each decrease of 1 log in RTE; *p* = 0.021).

## Discussion

Our study reports that pre-transplant thymic function predicts patient survival after transplantation. Indeed, patients with either low absolute number of RTE /mm^3^ or RTE frequency (%RTE) have a decreased life expectancy after kidney transplantation. This effect was observed in all sub-populations and remained significant after adjustment for major confounding factors. The same results were observed using TREC, another marker of thymic function, in a historical validation cohort. Among different causes of death, cancer-related death seemed particularly increased in patients with low RTE levels.

CD45RA^+^CD31^+^ CD4^+^ T cell appears to be relevant cellular phenotype to appreciate thymic activity (10). Although not identical with RTEs, it contains a RTE rich subset. Other markers of thymic function, such as the sj/beta-TREC ratio, are more complex and expensive and therefore clinically less useful. Many studies suggest that the determination of RTE is as efficient as TREC) to measure thymic activity (10). Moreover, we previously demonstrated strong correlations between TREC content in CD4^+^ T cells and frequencies of CD31^+^CD4^+^ T cells in end-stage renal disease patients (4). However, cytometric analysis of CD31^+^CD4^+^ T cells is standardized and results are not affected by homeostatic proliferation. Finally, measures are quickly available, warranting possible clinical application. Consequently, our results suggest that pre-transplant thymic function, as assessed by RTE, predicts death after kidney transplantation. Interestingly, we observed a similar association between premature death and pre-transplant thymic function assessed by TREC measurement in an independent validation cohort.

We previously reported that ATG-induced CD4 T cell lymphopenia is associated with increased morbidity (9, 11) and premature death after kidney transplantation (9). Concordant data also indicate that ATG exacerbates ESRD-associated immune senescence (12). Of note, a marked reduction in thymic output of RTE is observed in ATG-treated patients as compared with those having received α-CD25 mAb. CD4 T cell reconstitution is mainly dependent on thymus renewal (12) and patients with low post-transplant CD4 T cell levels after ATG also have low RTE concentrations (12). However, the association between RTE and survival was not influenced by the use of ATG. The present study extends our previous results concerning the deleterious effects of ATG-induced CD4 T cell depletion to a more general concept directly associating ESRD-associated immune senescence and death after transplantation.

All causes of death were increased in patients with low pre-transplant RTE levels. However, the association with cancer-related death seems critical. Indeed, patients with low pre-transplant RTE had a four-fold increase in death from cancer compared to those with higher thymic function and low RTE levels remained associated with cancer-related death even after adjustment on relevant factors. Previous studies reported higher incidence of cancer in KTR with low T CD4 cells levels (10–15). Nevertheless, CD4 T cell lymphopenia carries a low predictive value suggesting the need for a more precise diagnosis tool. Of note, we previously reported that pre-transplant TREC levels were predictive of cancer occurrence after transplantation (15). Associations of cancer occurrence with TREC and of RTE with cancer-related death strongly argue for the role of thymic dysfunction on post-transplant cancer development and progression. Thymic involution is also supposed to explain the increased incidence of cancers in elderly (16, 17) as a part of immune senescence-related disease. Nevertheless, few data really support this hypothesis; nevertheless, one study reported that TREC levels are lower in elderly patients with cancer compared to those without cancer (18). However, the rationale is mainly based on studies demonstrating that immunity against neo-antigens depends on CD4^+^ T cells recognizing a broad repertoire (19, 20). Other immune phenotypes have been described as being associated with cancer occurrence in KTR (20–22) and association of biomarkers including RTE may refine the prediction. In any event, low recent thymic emigrant levels characterize a transplant population at risk of cancer. Enhanced screening measures and considerations regarding immunosuppression are needed in these patients.

IL-7 is a cytokine implied in both T and B cell lymphopoiesis. In the thymus, IL-7 receptor stimulation promotes proliferation, differentiation and survival of the developing thymocytes. Recombinant IL-7 has been extensively studied for its potential to enhance immune recovery in different clinical setting of accelerated thymic involution (23). IL-7 has been shown to promote immune reconstitution both from thymus-independent homeostatic expansion of peripheral T cells and thymopoiesis in different clinical settings including idiopathic CD4 lymphopenia, septic shock, and cancer (24–28). Nevertheless, to date, no study assessed the potential efficacy of rhIL-7 in the ESRD-associated immune defect. Of note, better thymic function was not associated with an increased risk of graft loss suggesting that RTE expansion should not promote alloreactivity.

Our study has some limitations. Association does not preclude causality. As a consequence, we cannot assume that the association between RTE and death is not in fact due to a confounding factor. However, similar observations in other populations increase the likelihood of causality (29). Moreover, the robustness, the statistical independence, and the magnitude of our findings incite us to consider the result and its implications. Even when we included a large cohort, the studied event was hopefully rare. Consequently, associations may have been missed due to insufficient power. The replication cohort is quite different from the experimental one. Especially, the proportion of patients having received ATG is much higher which may have consequences regarding the effects of ATG on RTE. Moreover, thymic status was studied through TREC and not RTE. However, results were quite similar suggesting that our conclusions apply to different transplant settings.

Pre-transplant thymic predicts death after kidney transplantation. Patients with low pre-transplant levels should profit from specific screening and immunosuppressive strategies. Perspectives include the feasibility of thymus rejuvenation in ESRD-related immune senescence and their effects on patients' outcomes (Figure S6).

## Data Availability Statement

The raw data supporting the conclusions of this article will be made available by the authors, without undue reservation.

## Ethics Statement

The studies involving human participants were reviewed and approved by ethic committee of Franche-Comté. The patients/participants provided their written informed consent to participate in this study.

## Author Contributions

DD, JB, CC, EG, and TC designed the study concept and drafted the manuscript. TC, CC, JB, EG, and DD participated to acquisition of data and patient follow up. TC, CL, and PS participated in T cell subset analysis in patients. DD did statistical analysis. All authors saw, approved the final version of the manuscript, and approved the final manuscript.

## Conflict of Interest

The authors declare that the research was conducted in the absence of any commercial or financial relationships that could be construed as a potential conflict of interest.
