# Supplementary material for: Pre-transplant Thymic Function Predicts Is Associated With Patient Death After Kidney Transplantation
Source: Front Immunol. 2020 Jul 31;11:1653. doi: 10.3389/fimmu.2020.01653 (PMC7438875; doi:10.3389/fimmu.2020.01653)
Supplement: Supplementary file 1 [file Data_Sheet_1.doc]

**Table of contents:**

**Table S1: Correlation matrix between the different cell subsets**

**Table S2: Clinical characteristics of the experimental and validation cohorts**

**Figure S1: Fluorescent-activated cell sorting (FACS) analysis of recent thymic emigrant (RTE)**

**Figure S2A and S2B: Kaplan-Meier patient survival in ATG and non-ATG treated patients according to RTE quartiles**

**Figure S3: Kaplan-Meier death censored graft loss-free survival curves for patients according to a RTE quartiles**

**Figure S4: Correlation between RTE at transplant and RTE one year post-transplant**

**Figure S5: Kaplan-Meier death-free survival curves for patients according to a RTE threshold under the median value (132/mm^3^)**

ORLY study

The ORLY study is extensively described in Clinicaltrial.gov ([NCT02843867](https://clinicaltrials.gov/show/NCT02843867))

Briefly, ORLY-EST (9) is an observational prospective study including incident RTR in seven French transplant centres (Besançon, Clermont-Ferrand, Dijon, Kremlin-Bicêtre, Nancy, Reims, Strasbourg).

The main objective of this study is to describe interactions between immune status and post-transplant atherosclerosis. The study was performed after regulatory approval by the French ministry of health (agreement number # DC-2008-713, June 11^th^ 2009). The ethic committee of Franche-Comté has approved the study (2008). Patients enrolled in the ORLY-EST study gave their written informed consent. Clinical data were prospectively collected.

The study started in November 2008.

Eligibility criteria were as follows:

Inclusion Criteria:

1. Male or female patients aged over 18 years
2. Patients receiving a renal transplant
3. Absence of donor specific antibodies or negative cross-match
4. Patients able to understand the benefits and risks of testing
5. Patients gave written informed consent.

Exclusion Criteria:

1. Inability to understand the advantages and disadvantages of the study; psychiatric disorders judged by the investigator to be incompatible with the inclusion in the study.
2. Immunosuppressive therapy immediately prior to transplantation (including desensitization for HLA or ABO incompatible transplantation)
3. Cancer (except skin cancer) or malignant blood disease being treated; active infection; decompensated cirrhosis [patients had cancer and considered as cured or in remission, patients with virus infection of hepatitis B or hepatitis C and having no cirrhosis may be included].

Pre-transplant covariates analysed in ORLY study

- Age, gender, body mass index, diabetes, dyslipidemia, hypertension, smoking habit, a past history of cardiovascular events (CVE), chronic respiratory failure (CRF), and cancer.
- Dialysis mode (none, hemodialysis, or peritoneal dialysis), and its duration prior to transplantation.
- HLA mismatches for HLA-A, -B, and -DR loci. Other relevant immunological parameters such as, pre-transplant panel reactive antibodies (PRA) (0 *vs.* positive PRA at any level), transplant rank (first *vs.* second or more)

Definition of covariates

Past history of cardiovascular disease:

Coronary Heart Disease: Myocardial infarction, coronary revascularization including coronary artery bypass surgery or percutaneous transluminal coronary angioplasty, or typical history of angina with abnormal coronarography.

Stroke/Cerebrovascular Disease: Both nonhemorrhagic and hemorrhagic strokes or symptomatic extracranial artery stenosis resulting in carotid endarterectomy.

Abdominal Aortic or Lower Extremity Arterial Disease: Abdominal aortic repair, lower extremity revascularization via bypass surgery or angioplasty, lower extremity amputation

Chronic respiratory failure (CRF): diagnosis by a pulmonologist of respiratory failure whatever the origin of the pathology

**Table S1: Correlation matrix between the different cell subsets**

|  | | B | CD3 | CD4 | CD8 | monocytes | Inflammatory monocytes | NK | RTE |
| --- | --- | --- | --- | --- | --- | --- | --- | --- | --- |
| B | Correlation Coefficient Significance Level P n |  | 0,421 <0,0001 967 | 0,417 <0,0001 967 | 0,278 <0,0001 967 | 0,102 0,0018 879 | 0,069 0,0409 879 | 0,201 <0,0001 967 | 0,303 <0,0001 967 |
| CD3 | Correlation Coefficient Significance Level P n | 0,421 <0,0001 967 |  | 0,892 <0,0001 967 | 0,804 <0,0001 967 | 0,184 <0,0001 879 | 0,098 0,0032 879 | 0,288 <0,0001 967 | 0,629 <0,0001 967 |
| CD4 | Correlation Coefficient Significance Level P n | 0,417 <0,0001 967 | 0,892 <0,0001 967 |  | 0,464 <0,0001 967 | 0,217 <0,0001 879 | 0,105 0,0019 879 | 0,304 <0,0001 967 | 0,759 <0,0001 967 |
| CD8 | Correlation Coefficient Significance Level P n | 0,278 <0,0001 967 | 0,804 <0,0001 967 | 0,464 <0,0001 967 |  | 0,085 0,0102 879 | 0,061 0,0741 879 | 0,182 <0,0001 967 | 0,252 <0,0001 967 |
| monocytes | Correlation Coefficient Significance Level P n | 0,102 0,001 879 | 0,184 <0,0001 879 | 0,217 <0,0001 879 | 0,085 0,0102 879 |  | 0,642 <0,0001 879 | 0,221 <0,0001 879 | 0,065 0,0433 879 |
| Inflammatory  monocytes | Correlation Coefficient Significance Level P n | 0,069 0,0409 879 | 0,098 0,0032 879 | 0,105 0,0019 879 | 0,061 0,0741 879 | 0,642 <0,0001 879 |  | 0,195 <0,0001 879 | 0,000 0,9962 879 |
| NK | Correlation Coefficient Significance Level P n | 0,201 <0,0001 967 | 0,288 <0,0001 967 | 0,304 <0,0001 967 | 0,182 <0,0001 967 | 0,221 <0,0001 879 | 0,195 <0,0001 879 |  | 0,156 <0,0001 967 |
| RTE | Correlation Coefficient Significance Level P n | 0,303 <0,0001 967 | 0,629 <0,0001 967 | 0,759 <0,0001 967 | 0,252 <0,0001 967 | 0,065 0,0433 879 | 0,000 0,9962 879 | 0,156 <0,0001 967 |  |

**RTE: Recent thymic emigrants**

**Table S2**: Association between baseline characteristics and patient death (univariate and multivariate analysis)

|  | **Univariate analysis** | | **Multivariate analysis** | |
| --- | --- | --- | --- | --- |
|  | HR (CI 95%) | p | HR (CI 95%) | p |
| **Age** | **1.06 (1.04-1.08)** | **<0.001** | **1.04 (1.02-1.06)** | **<0.001** |
| **Male gender** | **2.90 (1.65-5.12)** | **<0.001** | **2.22 (1.22-4.12)** | **<0.001** |
| **BMI** | **1.08 (1.04-1.12)** | **<0.001** | **0.97 (0.93-1.04)** | **0.089** |
| **Diabetes** | **4.11 (2.64-6.32)** | **<0.001** | **2.50 (1.55-3.99)** | **0.001** |
| **Dyslipidemia** | 1.21 (0.71-1.56) | 0.253 |  |  |
| **Hypertension** | 1.32 (0.68-2.15) | 0.321 |  |  |
| **Current smoking** | 1.71 (0.88-4.21) | 0.156 |  |  |
| **Past history of CVE** | **1.68 (1.24-2.30)** | **0.011** | **1.35 (0.89-2.11)** | **0.102** |
| **Past history of cancer** | 2.31 (0.90-4.15) | 0.102 |  |  |
| **Chronic lung disease** | **2.41 (1.45-4.01)** | **<0.001** | **1.77 (0.92-3.25)** | **0.092** |
| **Dialysis** | 1.45 (0.80-2.66) | 0.186 |  |  |
| **Dialysis duration** | **2.09 (1.28-3.25)** | **0.003** | **1.59 (1.02-2.77)** | **0.041** |
| **Dialysis mode** | 1.13 (0.83-1.68) | 0.354 |  |  |
| **Deceased donor** | 1.98 (0.81-3.81) | 0.171 |  |  |
| **Donor age** | 1.01 (0.98-1.04) | 0.111 |  |  |
| **Cold ischemia time** | 1.05 (0.96-1.13) | 0.165 |  |  |
| **HLA mismatches** | 0.99 (0.89-1.15) | 0.421 |  |  |
| **PRA (>0)** | 1.31 (0.65-2.11) | 0.562 |  |  |
| **Delayed graft function** | 1.84 (0.79-4.01) | 0.337 |  |  |
| **Log RTE** | **4.22 (2.84-6.73)** | **<0.001** | **2.53 (1.54-4.39)** | **<0.001** |

**Table S3: Clinical characteristics of the experimental and validation cohorts**

|  | Experimental cohort  (n=967) | Validation cohort  (n=157) | p |
| --- | --- | --- | --- |
| Age (years) | 52+14 | 52+14 | 0.921 |
| Gender (% male) | 65% | 65% | 1 |
| Pre-transplant dialysis (%) | 91% | 91% | 1 |
| BMI (kg/m^2^) | 25.8+4.7 | 24.2+4.2 | 0.04 |
| Diabetes (%) | 21% | 21% | 1 |
| Percentage of immunized patients | 32% | 30% | 0.911 |
| Pre-transplant CMV exposure (%) | 55% | 77% | <0.001 |
| ATG use (%) | 30% | 69% | <0.001 |
