# Supplementary figures and images for: Pre-transplant Thymic Function Predicts Is Associated With Patient Death After Kidney Transplantation
Source: Front Immunol. 2020 Jul 31;11:1653. doi: 10.3389/fimmu.2020.01653 (PMC7438875; doi:10.3389/fimmu.2020.01653)

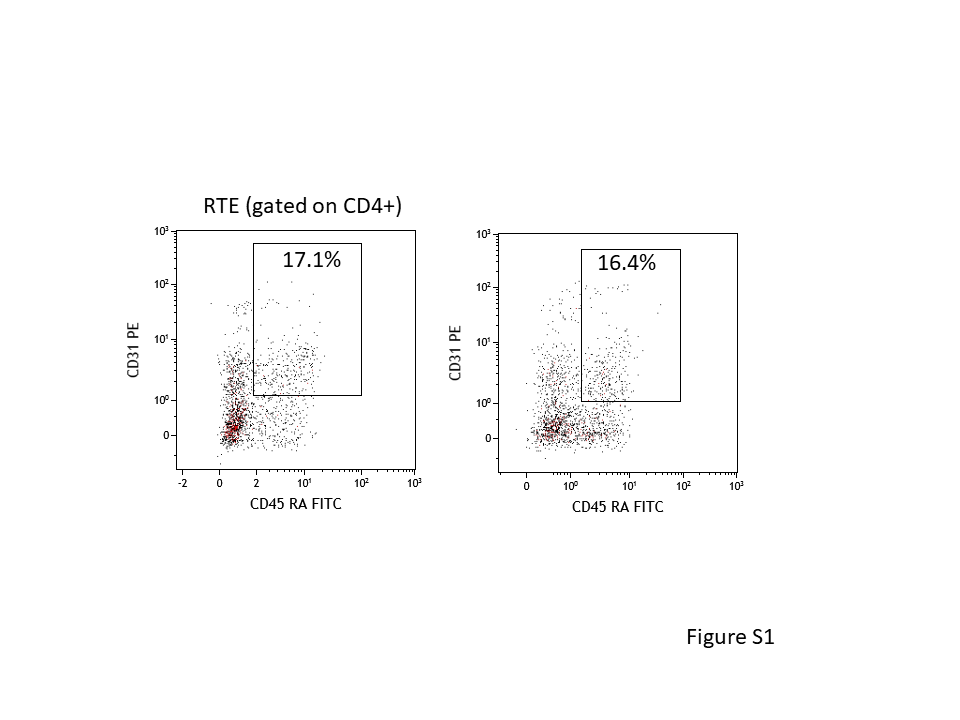

Supplement: Supplementary file 2 [file Image_1.TIF]

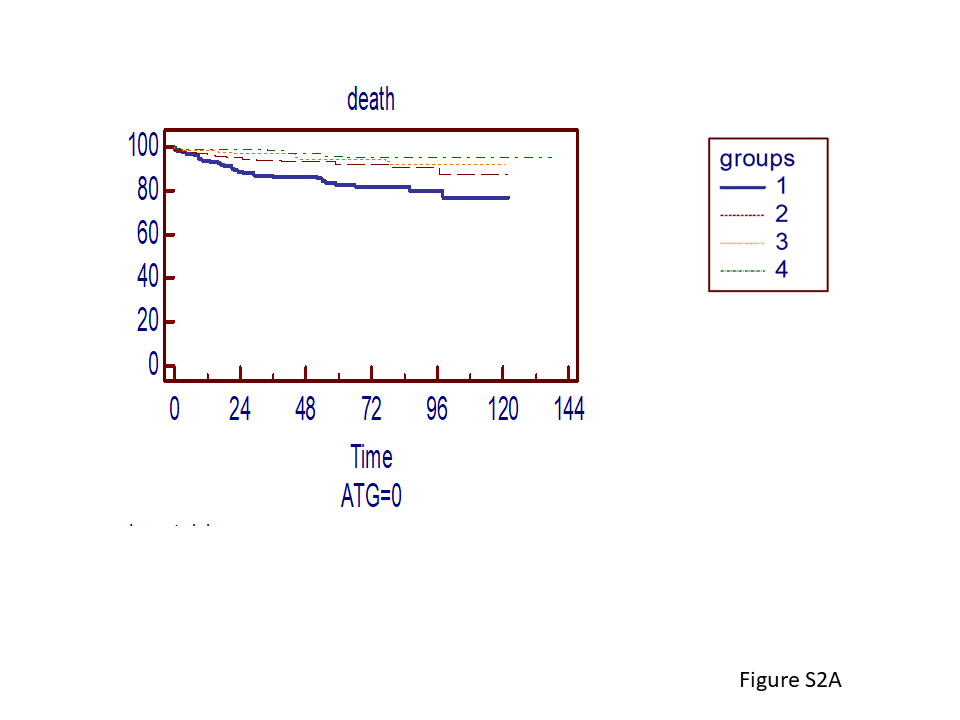

Supplement: Supplementary file 3 [file Image_2.TIF]

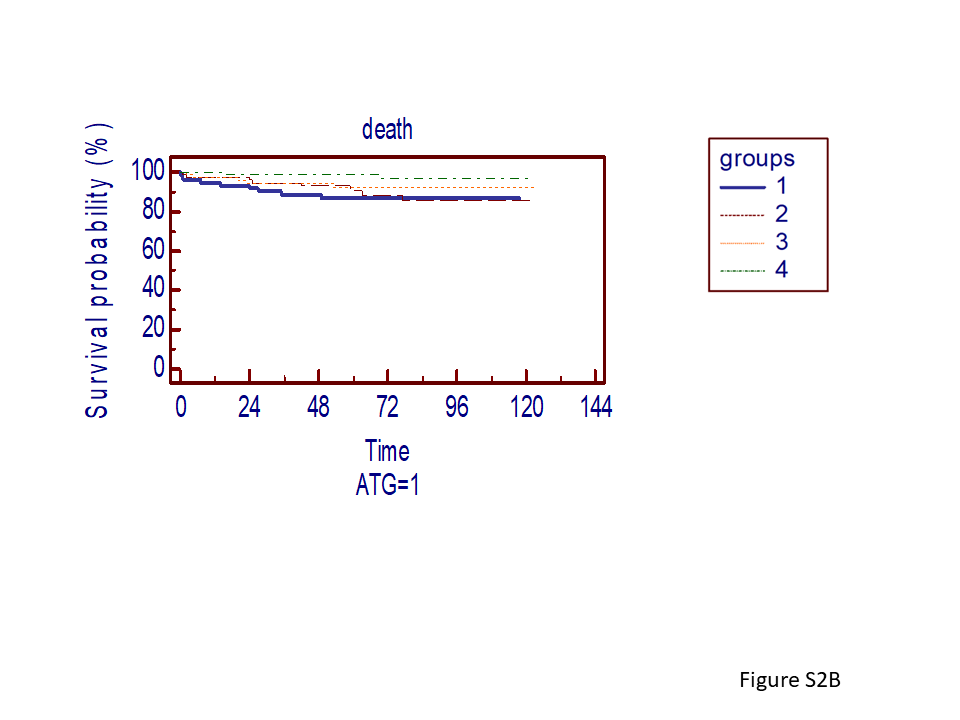

Supplement: Supplementary file 4 [file Image_3.TIF]

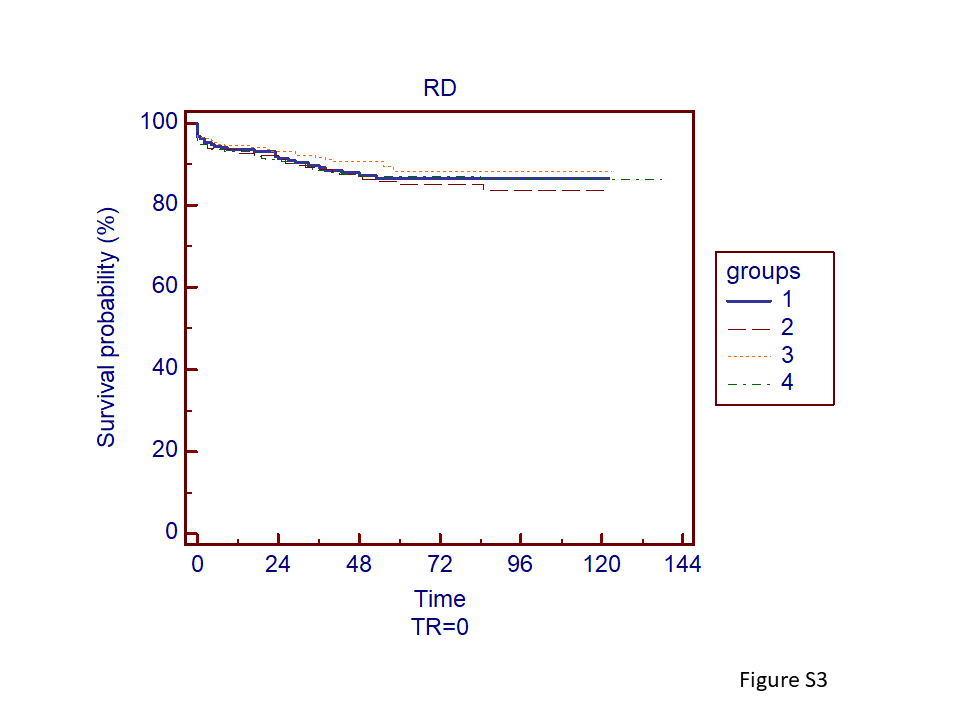

Supplement: Supplementary file 5 [file Image_4.TIF]

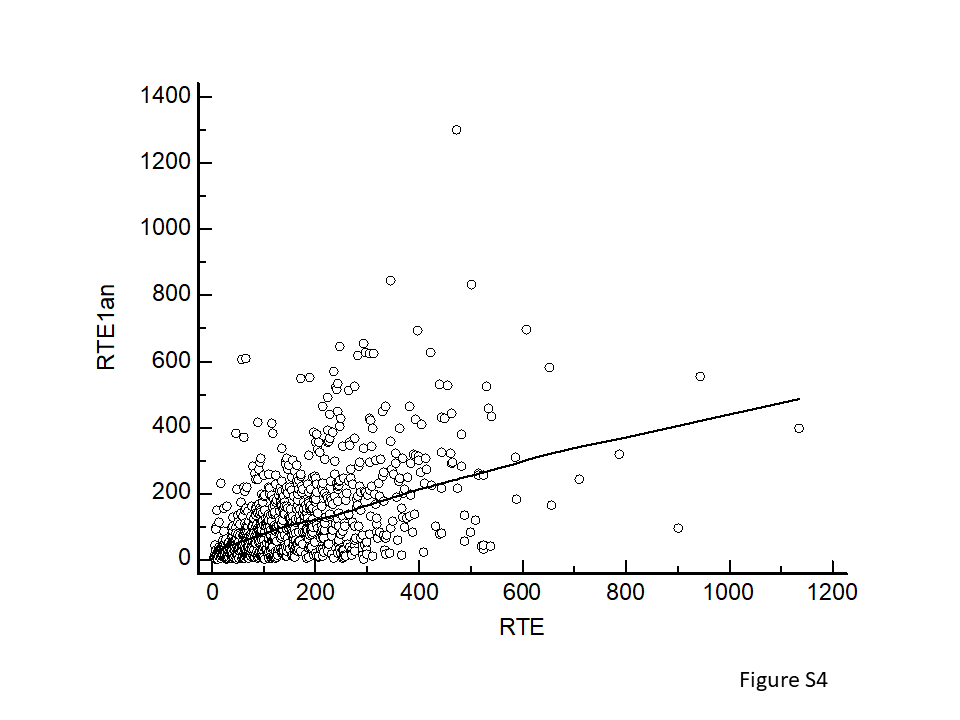

Supplement: Supplementary file 6 [file Image_5.TIF]

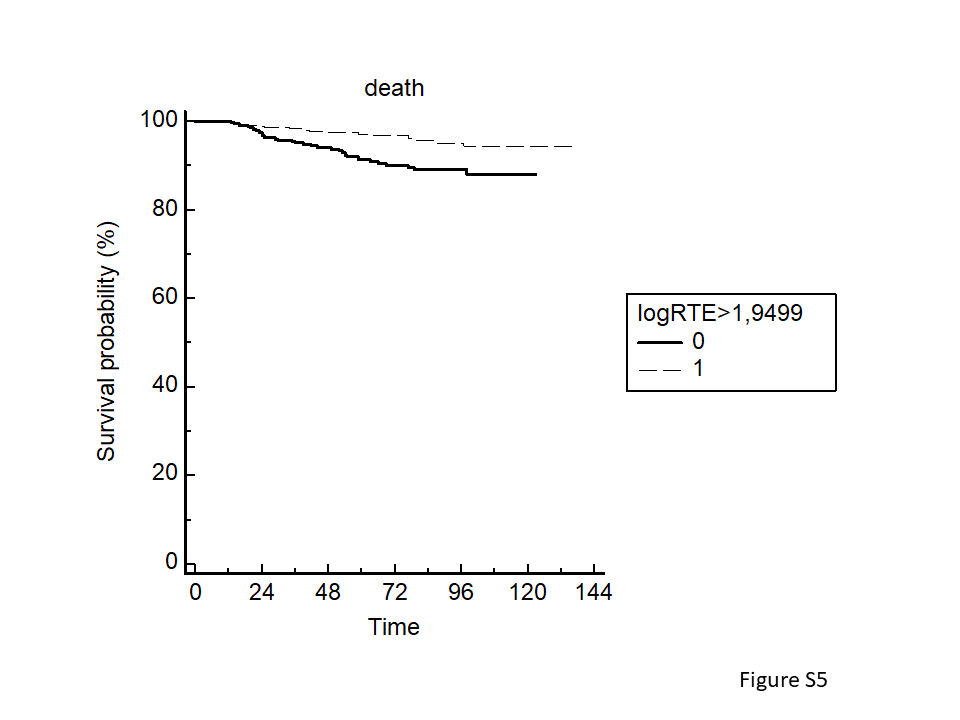

Supplement: Supplementary file 7 [file Image_6.TIF]

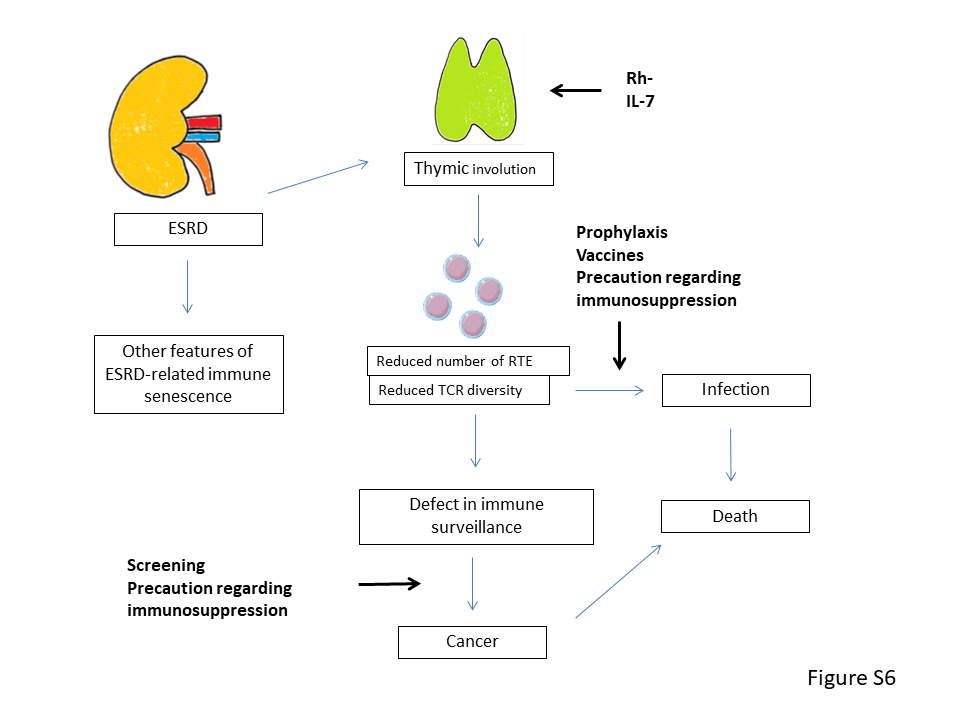

Supplement: Supplementary file 8 [file Image_7.TIF]
